# Remote-Controlled Microfluidic Platform for Real-Time Detection of Multiple Mycotoxins on Chip

**DOI:** 10.3390/foods15071180

**Published:** 2026-04-01

**Authors:** Jun Liu, Shiyu Zeng, Rashid Muhammad, Zhuoao Jiang, Gang Tan, Qi Yang, Binfeng Yin

**Affiliations:** 1 Suqian Product Quality Supervision and Inspection Institute, Suqian 223800, China; 2 School of Mechanical Engineering, Yangzhou University, Yangzhou 225127, China

**Keywords:** mycotoxins, remote-controlled, microfluidic platform, chemiluminescent (CL), colorimetric, food safety

## Abstract

Food safety requires real-time monitoring of mycotoxins in food, as food products contaminated with these toxins poses major threat to human health. In this study, we proposed a remote-controlled microfluidic platform (RCMP) integrated with chemiluminescent/colorimetric detection system for rapid, cost-effective and real-monitoring of multiple mycotoxins in real samples based on the indirect competitive enzyme-linked immunosorbent assay (ic-ELISA). The RCMP enabled sensitive and automatic detection of deoxynivalenol (DON), zearalenone (ZEA), and fumonisin B1 (FB1) in the range of 4–128 ng/mL, 1–32 ng/mL, and 0.5–16 ng/mL, respectively. The limits of detection (LOD) were 2.881 ng/mL for DON, 0.702 ng/mL for ZEA, and 0.470 ng/mL for FB1. In further validation, satisfactory recoveries between 93.57% to 108.47% with the relative standard deviations (RSDs) of 6.92–11.39% were obtained in beer samples. Overall, RCMP provides an automatic, high-throughput and cost-effective method for detection of DON, ZEA, and FB1 and can be confidently applied for monitoring in beer samples.

## 1. Introduction

Mycotoxin contamination causes substantial crop loss and poses a serious threat to food safety [1,2,3]. To date, approximately 500 different types of mycotoxins have been reported and identified in various food matrices such as cereals, dairy products, nuts, and fermented wines [4,5,6,7]. As the secondary metabolites produced by toxigenic fungi, these mycotoxins exhibited multiple acute and chronic toxicities along with teratogenicity, mutagenicity, and carcinogenicity, ultimately posing significant threat to human health [8,9,10]. According to statistical data from 2020, 68% of samples from foods were contaminated by at least two or more mycotoxins [11]. Various mycotoxins such as deoxynivalenol (DON), zearalenone (ZEA), and fumonisin B1 (FB1) frequently present in a single food matrix increase health risks due to synergistic toxic effects [12,13,14].

For human safety, different countries and regulatory organizations have established maximum limits of mycotoxins in food for human consumption. Different chromatographic methods such as ultra-performance liquid chromatography-tandem mass spectrometry (UPLC-MS/MS) and high-performance liquid chromatography (HPLC) are mostly used for the detections of these toxins [15,16,17]. These chromatographic methods demonstrate excellent selectivity, sensitivity and are regarded as the gold standard methods for quantitative analysis of multiple mycotoxins in different food samples [18,19,20]. However, the high-cost instruments, the demand for professional operators, and the complex pretreatment have limited their application in real-time food safety monitoring [21,22,23]. Therefore, developing simple, rapid, portable and cost-effective detection techniques for real-time monitoring of multiple mycotoxins is of great significance for food safety and a major trend in analytical chemistry [24,25,26]. Yin’s team proposed a portable lateral flow test strip based on SERS for the detection of AFB1, ZEA, and DON in corn samples, with a concordance rate of 87.18–93.60% compared to HPLC [27]. Zhang et al. developed a CRISPR/Cas12a-mediated unlabelled fluorescence biological sensing platform based on light copper nanoclusters to achieve sensitive detection of AFB1 with an LOD of 47.51 pg/mL [28]. Li’s team developed a multivariate signal-amplified photoelectrochemical aptasensor for detecting ZEA in the range of 0.1–20 ng/mL [29].

Microfluidic technology has attracted attention in quantitative analysis due to its low reagent consumption, rapid analysis, and high controllability [30,31,32]. These advantages make the microfluidic platform highly suitable for high-throughput, automated, and real-time detection [33,34,35]. Moreover, due to the easy integration of multi-step operations in microfluidic technology, various biochemical methods including ELISA, colorimetry, fluorescence, and chemiluminescence methods can be integrated onto a single microfluidic chip simultaneously to achieve simultaneous detection in multiple modes [36,37]. Based on this trend, researchers developed many microfluidic platforms based on biochemical analysis for real-time detection of mycotoxins [38,39]. For instance, Zou et al. proposed a portable visual/electrochemical dual-mode biosensing platform for simultaneous detection of ZEA and FB1, with a detection sensitivity of nanogram level [40]. Huang’s team designed a portable dual-mode paper chip integrating colorimetric and electrochemical luminescence signals to achieve ultra-sensitive detection of AFB1 with an LOD of 7.8 fg/mL [41]. These microfluidic platforms reduced reagent consumption and lowered detection costs; however, the preparation of reagents, signal capturing, and collection of waste liquid still required additional experimental equipments, limiting their automation and ease of operation for non-professionals.

**Figure 1:**
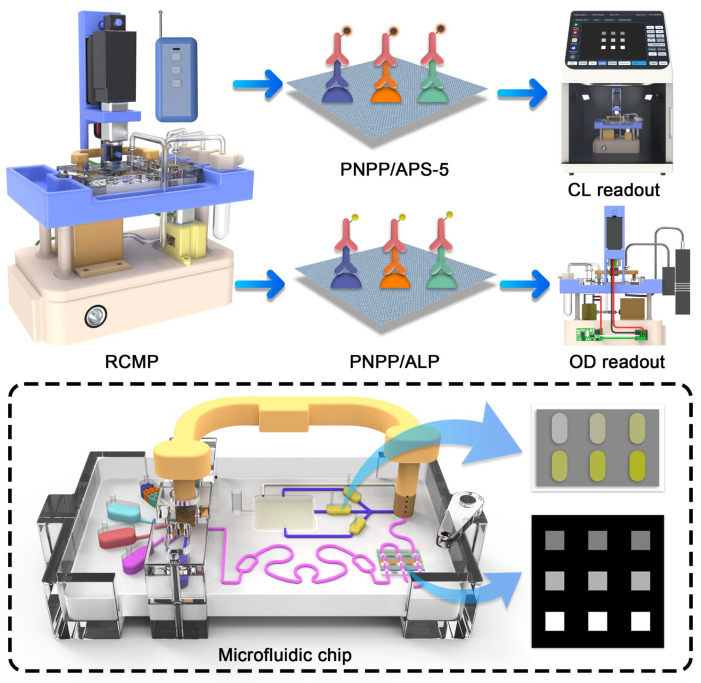
RCMP for real-time detection of multiple mycotoxins.

Herein, we proposed a remote-controlled microfluidic platform (RCMP) based on an indirect competitive enzyme-linked immunosorbent assay (ic-ELISA), integrating colorimetric and chemiluminescence dual-signal systems for real-time detection of DON, ZEA and FB1 on chip (Figure 1). In this platform, colorimetric and chemiluminescent methods were integrated into three capsule-shaped reaction reservoirs and reaction layer of the microfluidic chip, generating distinct capsule-shaped colorimetric and chemiluminescence arrays. The flow of the liquid in these reservoirs was controlled by a valve bank composed of two monomer valves. For efficient binding of antigens and antibodies in the fluid, a passive micromixer with flow resistance obstacles and circular-shaped microchannels was used. The semi-automatic detection on the chip was achieved by a set of autonomous-powered circuits, integrating a negative pressure pump for driving the fluid and a linear actuator for elevation of the valves. In addition, RCMP with miniaturization and convenience can be easily integrated into the Charge-coupled Device (CCD) camera system and Optical density (OD) reading system. This design achieved quantitative analysis of multiple mycotoxins in beer, lowering the detection cost, improving the detection efficiency, and reducing the need for professional operators, providing a new approach for semi-automatic and accurate real-time food safety analysis.

## 2. Materials and Methods

### 2.1. Materials and Instruments

To fabricate the microfluidic chip, polydimethylsiloxane (PDMS) with Sylgard 184 curing agent was purchased from Dow Corning Inc. (Midland, MI, USA). Meanwhile, the silicon film was sourced from Shanghai Shentong Rubber and Plastic Products Co., Ltd. (Shanghai, China). Immunological reagents of mycotoxins were purchased from Zhejiang Zhunce Biotechnology Co., Ltd. (Hangzhou, China), including standard solutions of DON, ZEA and FB1, ovalbumin (OVA)-conjugated antigens (DON-OVA, ZEA-OVA, FB1-OVA) and detection antibodies of DON, ZEA and FB1 (DON-Ab, ZEA-Ab, FB1-Ab). Bovine Serum Albumin (BSA) power was obtained from Tianjin Kangyuan Biotechnology Co., Ltd. (Tianjin, China). Detection antibody conjugated with alkaline phosphatase (IgG-ALP) and 4-nitrophenyl phosphate (PNPP) were supplied by Beijing Solarbio Technology Co., Ltd. (Beijing, China). Disodium [(4-chlorophenyl) sulfanyl] (10-methyl-9(10H)-acridinylidene) methyl phosphate (APS-5) was supplied by Changsha Xinlizhihe Technology Co., Ltd. (Changsha, China). Other analytical reagents with desired purity were purchased from Macklin Inc. (Shanghai, China).

The molds of chip and framework of RCMP were fabricated utilizing a YXIN-PRO LCD 3D printer manufactured by Yangzhou YIXIN 3D Technology Co., Ltd. (Yangzhou, China). A Putler plasma cleaner (Yantai, China) was employed for the efficient bonding of the PDMS layer. Colorimetric signals were analyzed with an XS11639 high-sensitivity fiber optic spectrometer from Shanghai Ruhai photoelectric Technology Co., Ltd. (Shanghai, China).

### 2.2. Design and Fabrication of Microfluidic Chip

The microfluidic chip with 83.5 mm long, 44 mm wide, and 16 mm thick, designed by SolidWorks 2016 consists of a PDMS layer, a reaction layer, a valve bank, and a set of brackets. The PDMS layer consists of upper and lower layers, where the upper layer was 5 mm thick and equipped with eight capsule-shaped reservoirs for the storage and incubation of reagents and the lower layer was 5 mm thick and served as a base layer equipped with a rectangular waste reservoir. The circular arc-shaped microchannels were utilized in the upper layers to connect these reservoirs. The reaction layer coated with OVA-conjugated antigens located in the middle of the PDMS layer was placed beneath a wave-shaped microchannel. Two monomer valves affiliated with the valve bank were used to control fluid flow, the valve 1 controlled the reagents in the six capsule-shaped reservoirs and the valve 2 controlled fluid flow to different reaction areas.

To manufacture the designed microfluidic chip, the molds of microfluidic chip, valve bank and brackets were fabricated utilizing a 3D printer. PDMS and curing agent were mixed at 10:1 ratio, degassed, poured into the molds, and then cured at 65 °C for 3 h in oven. After curing, the microchannel layer and base layer were peeled off from the mold with tweezers, and the burs were carefully trimmed off for subsequent bonding [30,32,39]. Subsequently, the upper and lower layers of the microfluidic chip were bonded via plasma treatment at a power of 200 W and an oxygen flow rate of 1.5 L/min.

### 2.3. Multiple Mycotoxins Detection on Chip

In the fabricated microfluidic chip, six capsule-shaped reservoirs with 57 μL volume capacity were used for storing multiple mycotoxins (including DON, ZEA and FB1), the capture antibodies (DON-Ab, ZEA-Ab, FB1-Ab), PBST, detection antibody IgG-ALP, APS-5 and PNPP, respectively. The lower layer was equipped with a rectangular waste pool with a volume capacity of approximately 357 μL. Initially, multiple mycotoxins and their corresponding capture antibodies were flowed into the reaction layer and three reaction reservoirs under negative pressure. Similarly, the detection antibodies were then injected under same condition. Finally, APS-5 and PNPP were pumped into the reaction layer and the reaction reservoirs. The CL and colorimetric signals were recorded by the CCD camera and the colorimetric analysis system. After the introduction of each reagent, PBST was used to clean the microchannel from the residual liquid of previous step, avoiding the mutual influence between the two systems.

### 2.4. Design and Fabrication of RCMP

The RCMP with 130 mm long, 73 mm wide and 172 mm high, consists of a framework, a linear actuator, a negative pressure pump, three tubes and a remote-controlled circuit. The framework was divided into three layers, where the first layer was a battery box storing a 12 V Lithium battery, the second layer consists of a waste tank with the volume capacity of 20 mL and four support columns for supporting RCMP structure, and the third layer consists of a bracket for linear actuator and a workbench for the microfluidic chip. The linear actuator mounted on the bracket, controls the lifting of the valve bank via a clamp. The negative pressure pump arranged on the second layer of the framework was connected to the microfluidic chip and waste tank via silicon pipe. Three tubes, which can store 2 mL reagent, are mounted on the workbench. The remote-controlled circuit consists of a wireless receiver, a switch control circuit, a DC connector, a charging port, a 12 V Lithium battery and a remote control, where the wireless receiver and switch control circuit, control the linear actuator and negative pressure pump. The remote control with three function keys UP, DOWN and PUMP transmits a 433 MHz signals, received by the wireless receiver and switch control circuit operating on the same frequency.

For the fabrication of the RCMP, the framework parts made of multiple-color resin were prepared through 3D printing process. After assembling the framework, the microfluidic chip was mounted on the workbench. Then the remote-controlled circuit with the negative pressure pump and linear actuator connected in parallel was assembled into the framework, where the pump was linked to waste tank and microfluidic chip via silicon pipe, and the linear actuator mounted on the dedicated bracket was connected to the valve bank via the clamp. Additionally, the tubes mounted on the workbench were connected to the microfluidic chip via silicon tube and needles.

### 2.5. Multiple Mycotoxins Detection on RCMP

On the fabricated RCMP, PBST, APS-5, and PNPP were injected into three reagents tubes, respectively, ensuring adequate reagent provision. The detection steps on chip were performed via remote control. The negative pressure pump was first activated by pressing “PUMP” to flow the reagents. The linear actuator was then activated to actuate the valve bank via “UP” and “DOWN”, thereby enabling the logical flow of the reagents. When APS-5 entered the reaction layer, CL array was captured by CCD camera system. Similarly, when PNPP entered three reaction reservoirs, the colorimetric signals were captured by colorimetric analysis system.

### 2.6. Pretreatments of Antigens on Reaction Layer

To incubate the antigens at 37 °C for 15 min in the reaction layer, DON-OVA, ZEA-OVA, and FB1-OVA were injected into a PDMS layer integrated with three linear microchannels, which was mounted on an 8 mm × 8 mm × 0.3 mm silicon film. After incubation, PBST was injected into the microchannels to wash away unbound antigens. Finally, PDMS layer was then removed from the silicon film to accomplish the immobilization on the reaction layer [42,43].

## 3. Results

### 3.1. The Construction and Characterization of Microfluidic Chip

**Figure 2:**
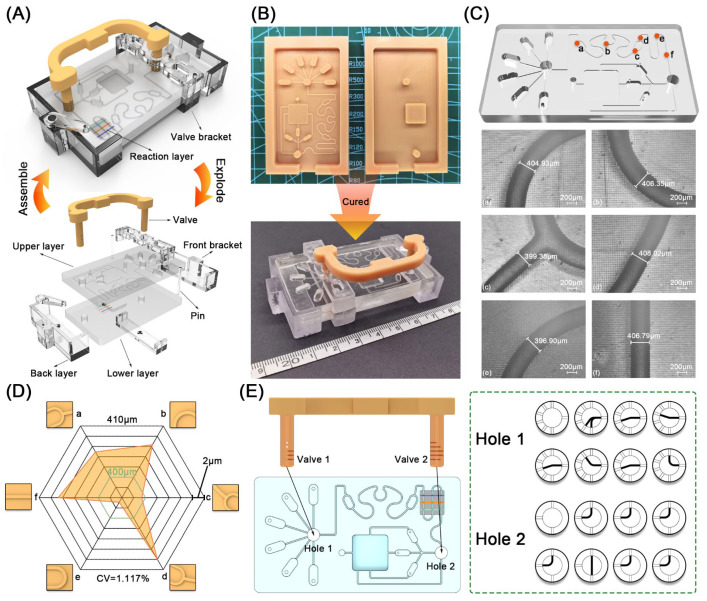
Characterization of the microfluidic chip. (**A**) The assembled and exploded microfluidic chip. (**B**) The fabricated molds and cured chip. (**C**) Local photographs (**a**–**f**) in six locations of microchannel. (**D**) Quantitative analysis of the microchannels. (**E**) Clock-shaped logical on-off states of the multi-stage valve bank.

Studying the structure of microfluidic chips is of great significance for achieving real-time detection of multiple mycotoxins. As shown in Figure 2A, the complete microfluidic chip consists of an upper layer, a lower layer, a reaction layer, a set of brackets and a valve bank. The upper layer integrated with microchannels was equipped with eight capsule-shaped reservoirs for reagent storage. The lower layer included a waste pool for the storage of the final reaction liquid. To fabricate the microfluidic chip, the molds of upper and lower layers were fabricated using an LCD 3D printer, followed by the curing of the chip (Figure 2B). After curing the PDMS layers, the brackets were mounted on the chip to reduce the vibration of chip. The valve bank integrating valve 1 and 2 was finally assembled into the microfluidic chip, where the valve 1 was inserted into hole 1, and the valve 2 into hole 2 (Figure 2E). Owing to the multi-stage microchannels integrated in the valve bank, the valve bank embedded in the hole at different stages exhibited eight clock-shaped logical on-off states. To assess the fabrication accuracy, the selected local microchannel (a–f) were measured using a metallographic microscope (Figure 2C). The corresponding results demonstrated excellent consistency with the design size (400 µm) and a coefficient of variation of 1.117% (Figure 2D), thereby indicating the high reliability of the printing process and PDMS replication.

### 3.2. Fluid Behavior Analysis in Micromixers

**Figure 3:**
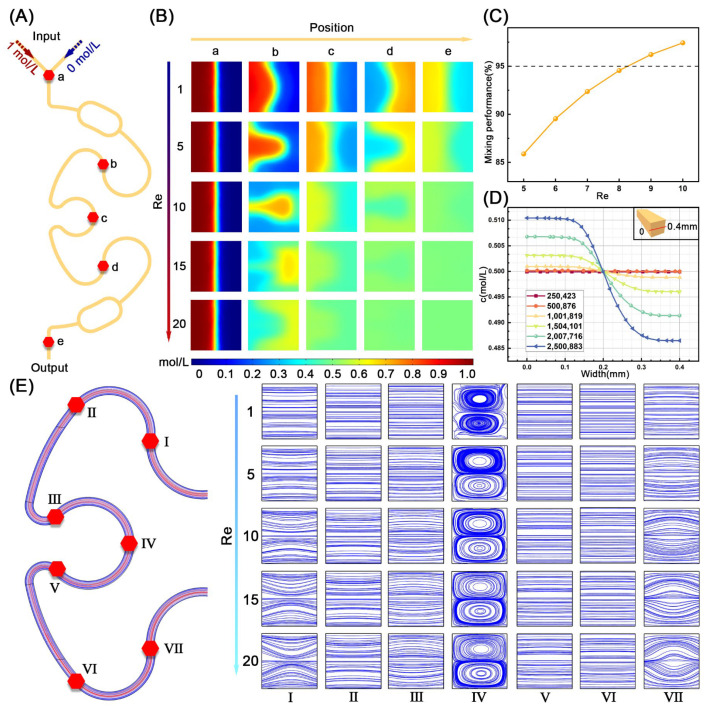
Fluid simulation of micromixer. (**A**) The designed arc-shaped passive micromixer. (**B**) The mixing performance of a–e at Re from 1 to 20. (**C**) The mixing efficiency of outlet at Re from 5 to 10. (**D**) Grid independence verification of the micromixer. (**E**) The velocity streamlines of the local cross-sections (I–VII) at Re from 1 to 20.

To ensure efficient binding between antigens and antibodies, we simulated and analyzed the mixing performance and liquid flow in the designed circular arc-shaped passive mixer. Specifically, the mixing performance of local microchannels from a to e in the micromixer was measured and evaluated using the COMSOL 5.6 software at different Reynolds numbers (Re) (Figure 3A). As shown in Figure 3B, when the Re and flow distance increase, the concentration streamlines in the cross-section become more curved and blurred, and the color approaches green, indicating an improvement in mixing efficiency. Since higher Re represents higher flow velocities and thus greater pressure, causing damage to the microchannels and leakage, the mixing efficiency at the outlet for a Re between 5 and 10 was further analyzed. As shown in Figure 3C, when the Re was greater than 8, the mixing efficiency has exceeded 95%, indicating excellent mixing efficiency [44,45]. Therefore, Re = 9 was selected as the optimal flow parameter. Additionally, grid independence verification was conducted by increasing the number of grids to ensure the reliability and authenticity of the simulation. In Figure 3D, as the number of grids increases, the maximum concentration change rate at the same position on the cut line at the outlet increases. When the grid number reached 2,007,716, the concentration variation rate was less than 1%, indicating the relatively stable mixing results. Therefore, 2,007,716 was selected as the optimum grid number.

By analyzing the mixing performance under the same Re, the liquid exhibited significant concentration changes after flowing through cross-sections at b, c, and d, indicating the generation of vortices. Therefore, to further study the flow situation of the designed micromixer, the velocity streamlines of the local cross-sections (I–VII) in the mixer were plotted under different Re. As shown in Figure 3E, as the Re increases, the streamlines in the cross-section no longer remain parallel to each other, and the fluid gradually becomes chaotic and turbulent, with two obvious Dean vortices appearing in section IV. These flow characteristics significantly enhance the fluid mixing performance.

### 3.3. The Dual-Mode Detection Process on Chip

**Figure 4:**
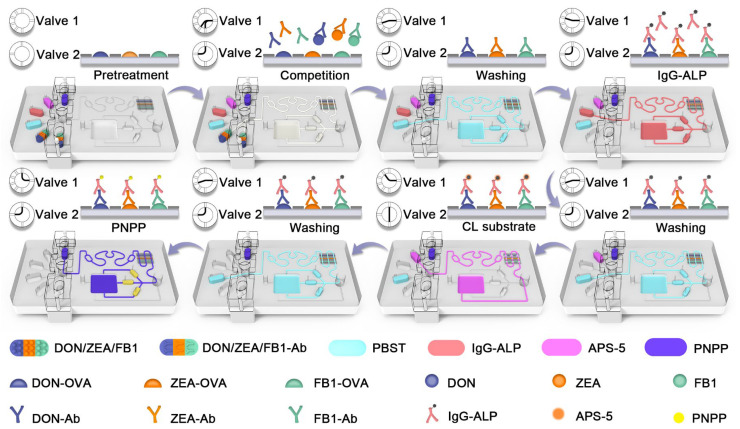
The dual-mode real-time detection process on the microfluidic chip.

To achieve real-time detection of DON, ZEA, and FB1, ic-ELISA integrating dual-mode signal systems was employed on the chip. As shown in Figure 4, six reagents including multiple mycotoxins, capture antibodies, PBST, IgG-ALP, APS-5, and PNPP were introduced into the corresponding reservoirs, respectively. DON-OVA, ZEA-OVA, and FB1-OVA were incubated on the reaction layer and reaction reservoirs in advance. Then, multiple mycotoxins and detection antibody were introduced into the reaction layer and three reaction reservoirs by the negative pump and the valve bank. The multi-stage valve bank controlled the valve 1 and valve 2, generating eight clock-shaped logical on-off states. In this step, capture antibodies were competitively bound by antigens and multiple mycotoxins. Similarly, IgG-ALP was introduced into the microchannel to bind with the capture antibodies immobilized on the reaction layer and reaction reservoirs. After that, APS-5 was pumped into the reaction layer and bypassed three reaction reservoirs via valve 2, thereby reacting with ALP to generate CL arrays. After that, PNPP was pumped into three reaction reservoirs, thereby reacting with ALP to generate colorimetric arrays. Additionally, during each detection step, enough PBST was provided by the tube in RCMP to wash away the residual reagents from the previous step, thereby avoiding cross-reaction. The detected signals were inversely proportional to the concentration of mycotoxins. Importantly, with the assistance of RCMP, the dual-mode detection can be carried out automatically.

### 3.4. The Construction of RCMP

**Figure 5:**
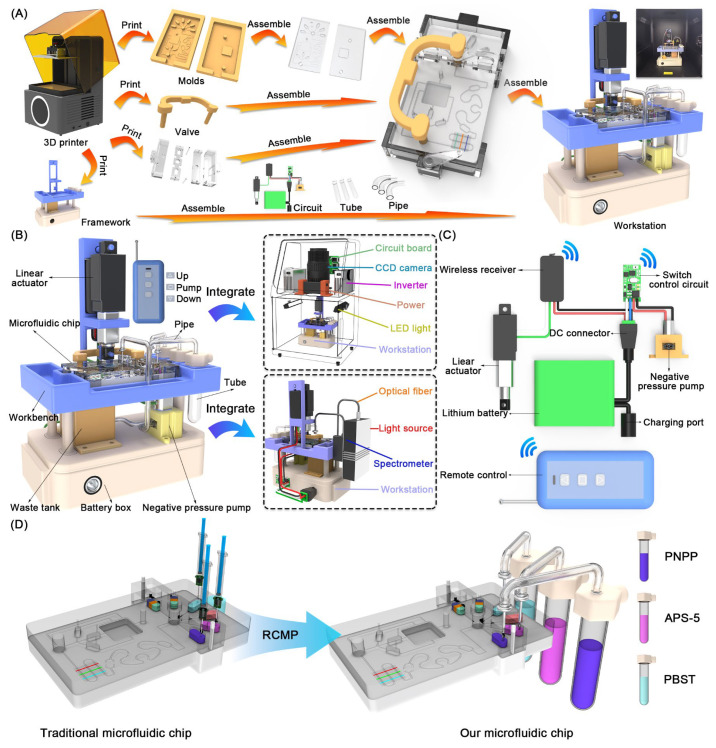
Characterization of RCMP. (**A**) The manufacture of RCMP. (**B**) RCMP integrating CL capture system and colorimetric capture system. (**C**) The remote-controlled circuit of RCMP. (**D**) The reagent replenishment function of the RCMP.

In order to detect multiple mycotoxins automatically, the RCMP was developed, achieving the remote control of the linear actuator and negative pressure pump. Specifically, The RCMP consists of a framework, a set of brackets, a valve bank, and molds, where the framework was equipped with a linear actuator, a negative pressure pump, tubes, and silicon pipes, and the molds curing PDMS layers, valve bank, and the brackets were utilized for the assemble of the microfluidic chip (Figure 5A). Due to the miniaturization and integration of RCMP, it can be easily integrated into a CCD camera system for capturing CL signals and a portable colorimetric signal capture system, consisting of optical fibers, a light source, a spectrometer, and a detector (Figure 5B). To achieve the remote control of RCMP, a remote-controlled circuit was mounted on the framework to activate the linear actuator and the negative pump automatically. As shown in Figure 5C, the remote-controlled circuit consists of a 12 V Lithium battery, a wireless receiver, a switch control circuit, a linear actuator, a negative pressure pump, and a remote control, where the battery was equipped with a Direct Current (DC) connector and a charging port, the linear actuator was controlled by the wireless receiver, and the negative pressure was activated by the switch control circuit. The wireless receiver can receive the 433 MHz signal sent by the remote control, thereby enabling the forward and reverse control of the motor in the linear actuator. Similarly, the switch control circuit can receive signals to control the start and stop of the negative pressure pump. Furthermore, compared with traditional microfluidic chips, RCMP enables real-time replenishment of detection reagents, including PNPP, APS-5 and PBST, demonstrating its strong real-time cyclic detection capability (Figure 5D).

**Figure 6:**
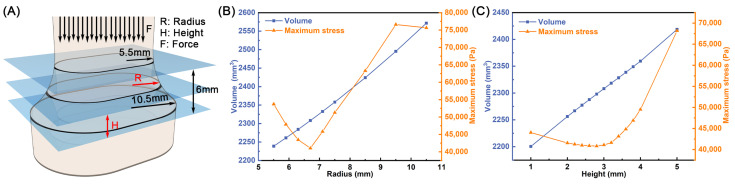
(**A**) Optimization of the support column. The volume and maximum stress of support column at different (**B**) radius and (**C**) height.

The support columns supporting the workbench in RCMP are the most crucial load-bearing component. By considering the applied load and volume, the designed support columns were simulated and optimized via finite element analysis of mechanical stress. As shown in Figure 6A, the design of the support columns consists of radius (R), height (H), and force (F), where R is the radius of the circle of the variable capsule structure, H is the height of the variable capsule structure from the lower plane, and F is the pressure generated by the structure above the supporting column of the RCMP. Since F remains constant during the operation of RCMP, R and H were optimized for the optimal combination. In Figure 6B, with the increase in R, the volume of the supporting column keeps increasing, which means more manufacturing materials will be consumed. Meanwhile, the maximum stress exhibited a trend of first decreasing and then increasing. By calculating the product of the volume and the maximum stress, we determined that 6.7 mm is the optimal value for R. Similarly, we also optimized H and finally selected 2.4 mm as the optimal value for H (Figure 6C).

### 3.5. Detection Performance of RCMP

**Figure 7:**
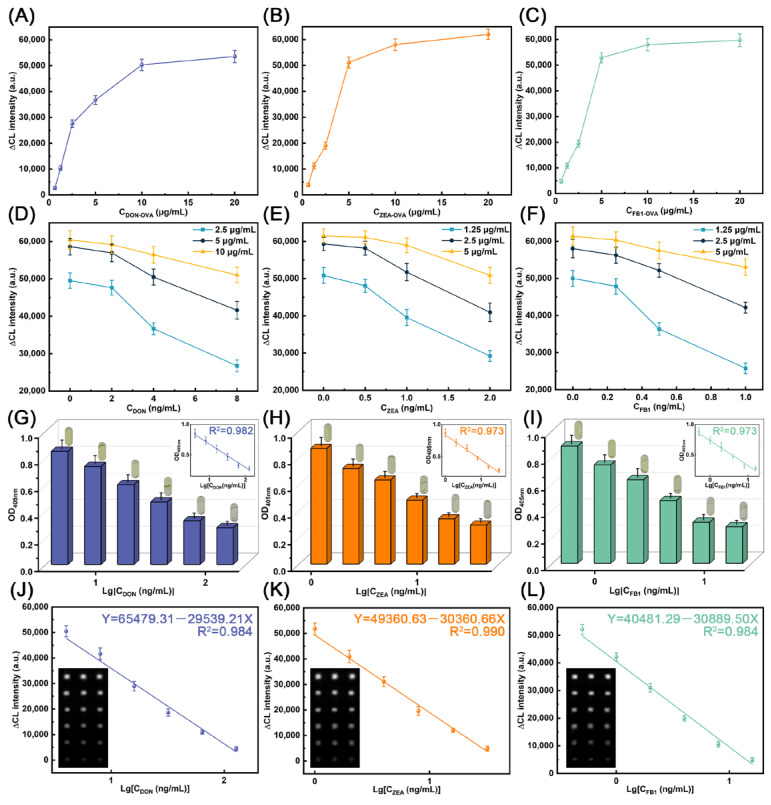
Detection performance of DON, ZEA, and FB1. Optimization of antigens including (**A**) DON-OVA, (**B**) ZEA-OVA, and (**C**) FB1-OVA. Optimization of capture antibodies of (**D**) DON-Ab, (**E**) ZEA-Ab, and (**F**) FB1-Ab. OD_405nm_ of (**G**) DON, (**H**) ZEA, and (**I**) FB1. Linear relationship between CL intensity and mycotoxins including (**J**) DON, (**K**) ZEA, and (**L**) FB1.

To achieve real-time detection on RCMP, detection reagents including antigens and antibodies were optimized based on CL signals. For DON-OVA, ZEA-OVA, and FB1-OVA, the corresponding antibodies were set to 20 μg/mL and the dilution ratio of the detection antibody was set at 1:1000. The CL intensity of antigens at concentration of 0.625–20 μg/mL was measured by CCD camera. As shown in Figure 7A–C, with the increase in antigen concentration, CL intensity also increases before saturation. Based on the detection limits of the instruments and the consumption amounts of the reagents, the concentrations of DON-OVA, ZEA-OVA and FB1-OVA were optimized to 10, 5, and 5 μg/mL, respectively. To optimize the concentration of capture antibodies, we selected the detection antibody of same concentration and the optimized antigens. In Figure 7D, DON-Ab was set to 2.5, 5, and 10 μg/mL to detect DON over the range of 0–8 ng/mL, respectively. These findings showed that when the concentration of DON was within the range of 0–2 ng/mL, the curve of 10 μg/mL of DON-Ab exhibited negligible changes, indicating the poor detection of the mycotoxin in the sample. When DON was over 8 ng/mL, the CL intensity of the curve of 2.5 μg/mL of DON-Ab will drop below 20,000, also indicating the hard detection at the corresponding concentration. Therefore, 5 μg/mL DON-Ab was selected as optimum concentration. Similarly, both ZEA-Ab and FB1-Ab were optimized to 2.5 μg/mL (Figure 7E,F).

**Figure 8:**
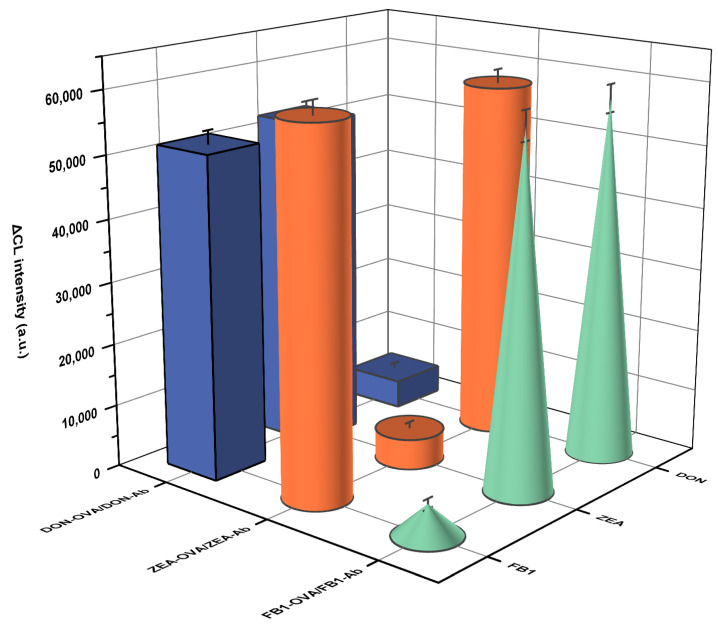
Specificity verification of DON, ZEA, and FB1.

Under these optimized concentrations of antigens and antibodies, RCMP integrated with dual-mode detection was employed to detect 4–128 ng/mL DON, 1–32 ng/mL ZEA, and 0.5–16 ng/mL FB1. As shown in Figure 7G–I, the concentration of mycotoxins exhibits a linear correlation with OD_405nm_ with regression coefficients (R^2^) of 0.982, 0.973, and 0.973, respectively. Meanwhile, visible color changes in the capsule-type reservoirs could be observed by the naked eye. These results show that the colorimetric method can be used for the accurate quantification of multiple mycotoxins. The CL detection method was also utilized for the real-time detection of DON, ZEA, and FB1. As shown in Figure 7J–L, the logarithms of the concentrations of DON, ZEA, and FB1 also exhibited a strong linear relationship with the CL intensity. According to the three-time signal-to-noise ratio (3S/N), the calculated LODs were 2.881 ng/mL, 0.702 ng/mL, and 0.470 ng/mL for DON, ZEA, and FB1, respectively. To verify the specificity of RCMP, the antigen-antibody detection groups including DON-OVA/DON-Ab, ZEA-OVA/ZEA-Ab, and FB1-OVA/FB1-Ab, were used to detect the individual mycotoxins including DON, ZEA and FB1. As shown in Figure 8, in addition to the specific targets, the non-specific groups all exhibited significant CL signals, demonstrating the excellent specificity of RCMP. Furthermore, RCMP was utilized for the quantification of mycotoxins in beer to verify its practical application in real-world scenarios. As shown in Table 1, recovery between 93.57% to 103.59% with RSD less than 9.37% was obtained after spiking beer samples with 4.5–72 ng/mL of DON. Similarly, RCMP was utilized to detect ZEA and FB1 in spiked beer, where the recovery of ZEA ranged from 99.36% to 104.18% with the RSD of 6.92–9.37%, and the recovery of FB1 ranged from 95.62% to 107.56% with the RSD of 7.39–11.39%. This is comparable to the recovery rate of 96.39–104.52% achieved by HPLC, further validating the powerful ability of RCMP to detect multiple mycotoxins in real samples. We also compared the work of RCMP with that of other recent researchers. As shown in Table 2, while maintaining a low LOD and stable recovery rate, our RCMP demonstrates an advantage in the number of target substances it can detect, indicating its reliability in real-world applications.

## 4. Conclusions

In this study, we successfully developed an RCMP integrating dual-mode signal system for the detection of multiple mycotoxins on chip based on ic-ELISA. By integrating the remote-controlled circuit, the RCMP enabled real-time and automatic detection of DON, ZEA, and FB1, where DON ranges from 4 to 128 ng/mL, ZEA ranges from 1 to 32 ng/mL, and FB1 ranges from 0.5 to 16 ng/mL. Significantly, the dual-mode chemiluminescent/colorimetric signal readout assisted RCMP in flexibly detecting multiple mycotoxins in complex food matrices, effectively reducing the false positive/negative rate and enhancing the practicality and reliability. In beer samples, RCMP demonstrated satisfactory results with a recovery rate ranging from 93.57% to 108.47%. Meanwhile, it also exhibited desirable LODs for multiple mycotoxins in comparison with other detection methods. These findings substantiate the practical applicability of our RCMP in real-world scenarios. More importantly, this RCMP can further integrate different functionalized microfluidic chips, thereby expanding to high-throughput screening scenarios for multiple target substances such as veterinary drug residues, heavy metals, and biomarkers. It provides a universal and scalable platform-based solution for rapid and intelligent multi-component detection on-site.

## Figures and Tables

**Table 1:** Recovery of DON, ZEA, and FB1 in beer samples.

Mycotoxins	Spiked Level (ng/mL)	Recovery of RCMP (%)	RSD (%)	Recovery of HPLC (%)	RSD (%)
DON	4.5	95.16	8.51	97.59	7.47
	9	102.38	7.75	101.47	2.51
	18	98.07	7.02	99.21	5.32
	36	103.59	6.92	102.15	6.21
	72	93.57	9.37	96.39	5.25
ZEA	1.5	102.69	7.43	99.47	7.58
	3	108.47	7.93	104.52	6.59
	6	99.36	9.92	101.26	8.15
	12	103.62	8.46	102.49	7.67
	24	104.18	10.34	99.52	3.22
FB1	0.75	106.72	7.81	102.49	9.79
	1.5	95.62	9.49	97.42	7.59
	3	107.56	8.54	101.71	6.31
	6	102.48	11.39	99.29	3.69
	12	98.49	7.39	101.17	8.18

**Table 2:** Comparison of detection index for mycotoxins of different methods.

Method	Mycotoxins	LOD	Real Sample	Recovery (%)	RSD (%)	References
BA-COF	ZEA	0.26 μg/kg	Maize, Millet, Oat, Wheat	89.3–106	1.8–5.4	[46]
MBs	ZEA AFB1	4.91 μg/kg 0.17 μg/kg	Oil	96.5–110.7	<10.4	[47]
LED-IF	AFB1 OTA ZEA	0.33 ng/mL 1.80 ng/mL 28.2 ng/mL	Standard samples	-	-	[48]
CS-UCNPs	DON	0.1 ng/mL	Corn, wheat	94.74–104.81	4.49–7.17	[49]
MOF	ZEA	0.05 ng/mL	Maize	86.3–103.6	7.6–10.1	[50]
RCMP	DON ZEA FB1	2.881 ng/mL 0.702 ng/mL 0.470 ng/mL	Beer	93.57–108.47	6.92–11.39	This work

## Data Availability

The original contributions presented in this study are included in the article. Further inquiries can be directed to the corresponding author.

## Abbreviations

The following abbreviations are used in this manuscript:

RCMP	Remote-controlled microfluidic platform
DON	Deoxynivalenol
ZEA	Zearalenone
FB1	Fumonisin B1
ic-ELISA	Indirect competitive enzyme-linked immunoassay
LOD	limit of detection
CL	Chemiluminescent
HPLC	High-performance liquid chromatography
LC-MS	Ultra-performance liquid chromatography-tandem mass spectrometry
PDMS	Polydimethylsiloxane
OVA	Ovalbumin
IgG-ALP	Detection antibody conjugated with Alkaline phosphatase
SA	Streptavidin
PNPP	4-nitrophenyl phosphate
BSA	Bovine Serum Albumin
APS-5	Disodium [(4-chlorophenyl) sulfanyl] (10-methyl-9(10H)-acridinylidene) methyl phosphate
Ab	Antibody
RSD	Relative standard deviation
CV	Coefficient of variation
Re	Reynolds numbers
CCD	Charge-coupled Device
OD	Optical density
